# Mobile alert app to engage community volunteers to help locate missing persons with dementia

**DOI:** 10.1371/journal.pone.0254952

**Published:** 2021-07-19

**Authors:** Noelannah Neubauer, Christine Daum, Antonio Miguel-Cruz, Lili Liu

**Affiliations:** 1 School of Public Health Sciences, Faculty of Health, University of Waterloo, Waterloo, Ontario, Canada; 2 Department of Occupational Therapy, Faculty of Rehabilitation Medicine, University of Alberta, Edmonton, Alberta, Canada; University of Ottawa, CANADA

## Abstract

The prevalence of persons living with dementia and at risk of going missing is rising. In this study, we engaged persons living with dementia, care partners, police services, search and rescue organizations, and health and social service providers to develop Community ASAP, a mobile alert system that engages community citizens, as volunteers, to look out for persons with dementia reported missing. We completed three phases of development and evaluation of the usability and functionality of the alert system with stakeholders in three Canadian provinces. In this paper we describe features of the Community ASAP and the findings of these evaluation phases.

## Introduction

The number of missing person incidents involving persons living with dementia has been on the rise in recent years [1]. The consequences associated with missing persons with dementia include injuries, exposure to extreme temperatures, dehydration, and death [2]. If not found within 24 hours, up to half of individuals who get lost succumb to serious injury or death [3]. Therefore, response time is critical. Research indicates that members of a community, such as neighbours and shop owners, can complement search-and-rescue efforts by being ‘extra eyes’ on the lookout for persons living with dementia reported missing [4].

The Silver Alert program, an initiative first launched in the United States in 2006, is a publicly funded notification system that broadcasts information about missing persons with dementia or other mental disabilities, to engage members of a community to assist in the location of missing persons [5]. All but five US states have this program [5]. The perceived successes of this program have led to public pressure to implement a silver program in Canada [6]. In British Columbia, a citizen-funded Silver Alert program [7] was founded in response to a case that involved a person with dementia who had gone missing and has never been found [8]. In 2017 and 2018, two provinces, Alberta [9] and Manitoba [10], amended their missing persons acts to include ‘silver alert’. The amendment of Manitoba’s Missing Persons Act refers to "silver alert" as "a broadcast messaging system that disseminates information to the public through the media and other means as soon as practicable after a vulnerable person or another adult with a cognitive impairment goes missing in an effort to safely recover the adult" (1.1(1), p.1). It also states the “Police and broadcasters may collaborate to activate silver alerts, a police service may enter into an arrangement or agreement with one or more broadcasters and any other partners that the police service considers appropriate" (1.1(2), p.1). The amendment however does not actually change existing protocols for missing persons living with dementia in the province.

In February 2019, Petition no. 421–03207 was presented to the House of Commons with 1034 signatures for Government of Canada to create a national Silver Alert program [11]. The Government tabled a response in February 2019 acknowledging this issue and stating its commitment to improving the lives of older Canadians living with dementia and their caregivers, demonstrated through the National Dementia Strategy released in June 2019 [12]. Canada’s National Public Alerting System (NPAS), also known as ‘Alert Ready’, is a Provincial-Federal-Territorial initiative used to alert Canadians of all-hazards (e.g., tornados, chemical spills) through radio, cable and satellite television, email, text services on compatible wireless devices. Amber Alert is used for missing children, typically due to abduction. The number of vulnerable seniors who go missing are higher than natural and other types of disasters, and are not related to crime generally. Therefore, Alert Ready and Amber Alert would quickly cause alert fatigue and not be effective to use in situations of missing seniors.

Publicly funded silver alert programs in the United States also have limitations. They are inconsistently implemented across jurisdictions; for example, the initiation of alerts and their duration vary across states. Alerts are often broadcasted using television and road signs. Yet, these mediums have progressively less reach as more people access content via their mobile devices. There is risk of declining sensitivity to media due to alert fatigue [13]. An ideal solution for Canada would be programs that engage local communities, cross provincial boundaries, and minimize alert fatigue by being location specific. Programs should also respect the rights to self-determination and privacy among persons living with dementia.

To our knowledge, there is no alert app that exist in Canada. Nor is there a localized alert system within Canada that allows citizens, businesses and first responders to work together to locate a missing person. The purpose of this project was to develop and evaluate the accuracy and usability of a mobile application, the first of its kind, called Community ASAP, that allows first responders to alert community volunteers of missing older vulnerable adults.

In this paper, we provide a description of the alert app that we co-designed with representatives of end-users. We describe the features of this alert app and the three phases of the iterative design and evaluation process that involved 70 stakeholder participants. Finally, we describe the process of evaluating the accuracy and usability of the alert app we designed.

## Methods

### System overview

**Fig 1:**
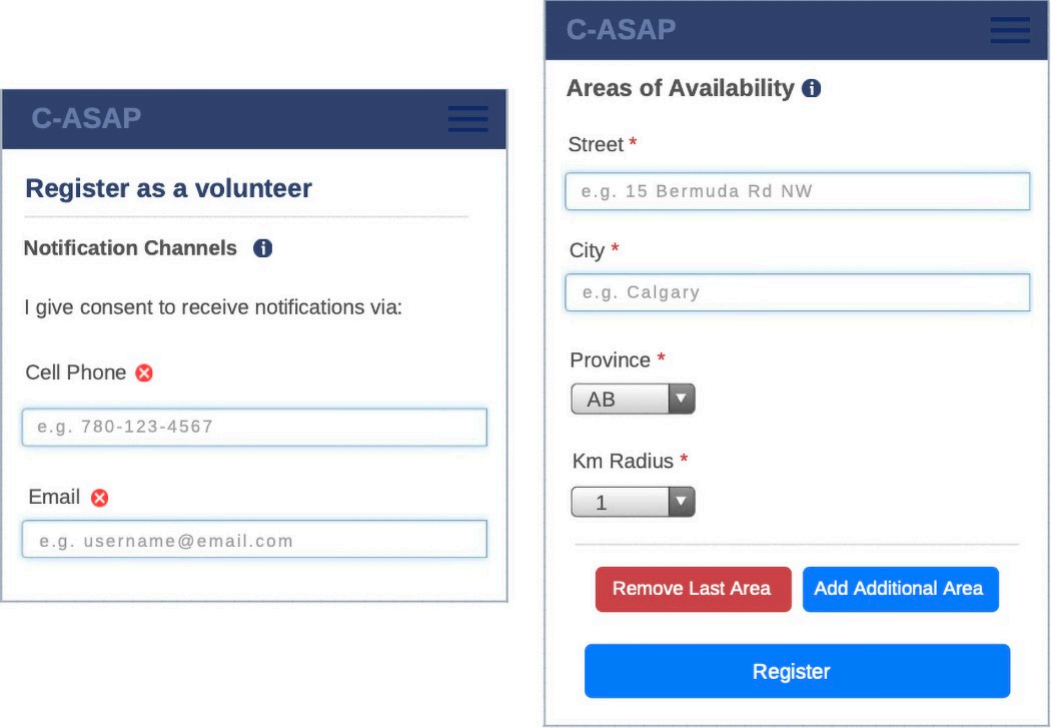
Screenshots of registration feature for volunteer.

Community ASAP is a localized area alert system for missing persons with dementia that allow volunteers, local businesses and police services to work together to recognize and locate a missing person. Community ASAP is available online as a mobile application and is compatible with Apple iOS and Android operating systems. Community volunteers receive alerts based on their geographic preferences and can choose the specific radii for up to five specific locations: 1, 3, 6, 12, 25 km (Fig 1). Volunteers would typically choose their home and work addresses as specific locations. Volunteers are community citizens who are willing to ‘keep an eye out’ for a missing person; they’re instructed not to actively search for missing persons living with dementia, only to report if they see a person matching the description of a person reported missing. Volunteers register with Community ASAP by providing their full name, email address and mobile phone number, areas of availability, i.e., specific location(s) with preferred radii, and alert preferences (SMS, email, downloaded app) (Fig 1). Volunteers can view tips and resources on how to report or respond when they see a person reported missing within a radius of their location(s). The tips and resources were developed in collaboration with the Calgary Coordinated Response to Missing Seniors, who work closely with persons living with dementia.

A Community ASAP Coordinator initiates the alert, and this role is typically adopted by police services. When a person with dementia is reported missing, the coordinator creates a profile that includes the following information: first and last names, nickname, age, height, weight, eye color, hair color, location last seen, description of clothing, level of mobility and mobility devices, and favourite locations they like to visit.

**Fig 2:**
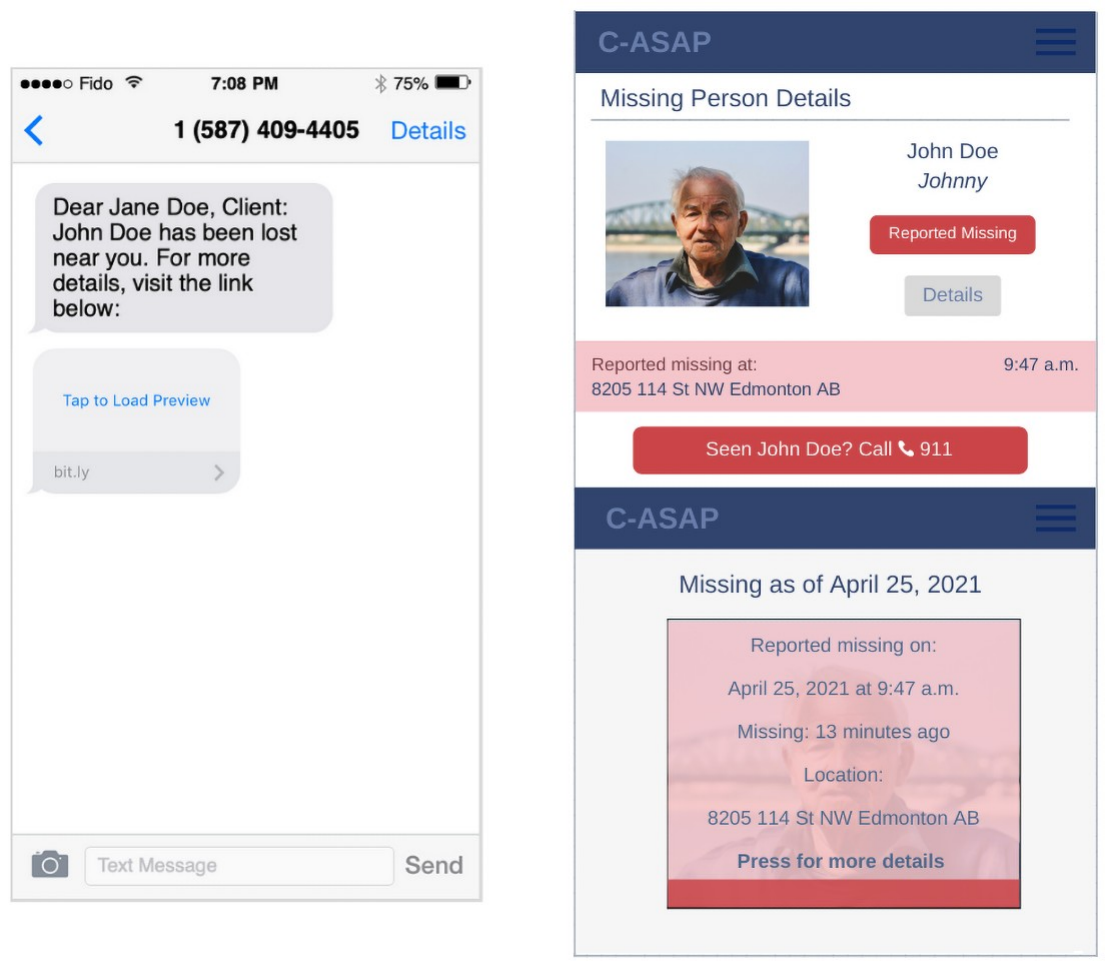
Screenshots of alert about mock missing person sent to Community ASAP volunteers using stock images.

The Community ASAP alert systems process begins when a care partner or staff of a care facility call the local emergency number (i.e., 911 in Canada) to report a missing person. The emergency dispatcher collects the intake information and forwards internally, where a patrol car is then dispatched. The police officer visits the last known address, interviews the person who made the report, and initiates the ground search. After crime is ruled out by police and if the person is not found, the Community ASAP Coordinator sends a missing person alert to volunteers Community ASAP (Fig 2). The timing associated with ruling out crime and initiating an alert is at the discretion of police and varies depending on the situation. This is done in conjunction with current standard practice of sending alerts using social media (Twitter, Facebook). Volunteers keep a look out for the missing person and if they see the missing person, they use the feature embedded within the Community ASAP app to contact the emergency number or call the specified emergency number directly. Police are dispatched to confirm the report and assist the missing person. Once confirmed, the Coordinator sends a closing report notifying volunteers that the missing person was located. Details about the person and the alert are permanently removed from the Community ASAP system and from volunteers’ browser history through the community coordinator.

### System and study design

The design used an iterative process [14] consisting of three phases and six development and evaluation phases over one year (Table 1). In Phase I, we developed the first prototype of Community ASAP based on three consultations with one stakeholder group. In Phase II, we tested an improved version with four stakeholder groups, each subsequent group tested an improved version. In Phase III, the final prototype incorporated suggestions from the previous phases. We used a multi-method approach to assess the accuracy and usability of Community ASAP during each phase. In this paper, we report the accuracy and usability in the final phase as improvements were made to the system and the interface after each phase. Participants were recruited across three Canadian provinces using snowball sampling [15] and included persons living with dementia, community organizations, police services, and volunteer search and rescue. Ethics approval was received from the University of Alberta Research Ethics Board 2 (Pro00078537). Written consent was acquired from each participant prior to their involvement in the study.

**Table 1:** Community ASAP overview.

Community ASAP Overview			
	Phase I	Phase II	Phase III
**Purpose(s)**	Pilot *web-based system* accuracy and usability.	Evaluate *web-based system* accuracy and usability.	Evaluate *mobile application* accuracy and usability.
		Understand police and search and rescue process related to missing persons incidents and determine how Community ASAP can be integrated.	
**Location**	Edmonton, Alberta	3 Canadian metropolitan police services (Alberta, Ontario) and 1 volunteer search and rescue organization (British Columbia)	Virtual (online), with representation from sites in Phases I and II
**Participants**	10 graduate students and 2 older adult advisors	45 persons living with dementia, family care partners, police and volunteer search and rescue members, health and social care providers	13 older adult advisors, persons living with dementia and family care partners, police and volunteer search and rescue members, health and social care providers
**Activities**	3 in-the-field scenarios	3 simulated (in-office) and 1 in-the-field scenarios. These were conducted one at a time and improvements were made after each test.	1 simulated (in-office) scenario with 130 alerts
**Evaluation**	Tracking accuracy and timing of alerts and messages sent and received *(Alert Tracking form)*	Ease of use, organization, aesthetics *(Website Usability Questionnaire)*	Tracking accuracy and timing of alerts and messages sent and received *(Alert Tracking form)*
	Ease of use, organization, aesthetics *(Website Usability Questionnaire)*	User challenges, frustrations, suggestions for improvement *(Participant observation*, *focus groups)*	Ease of learning and use, helpfulness and ease of problem-solving, aesthetics and affective responses, organization, control and efficiency *(Website Usability Questionnaire)*
	User challenges, frustrations, suggestions for improvement *(Participant observation*, *focus group)*	Police and volunteer search and rescue processes to determine how Community ASAP can be integrated to current systems *(Focus groups)*	User experiences and suggestions for improvement *(Focus group and email responses to open-ended questions)*
**Analysis**	Descriptive statistics (*Alert Tracking form*, *Website Usability Questionnaire)*		
	Conventional content analysis (*Participant observation*, *focus groups*, *emails)*		

In all study phases and locations, with the help of community research partners, we created scenarios to simulate the events that transpire during a missing person event. In these scenarios, participants assumed the key roles in the Community ASAP system including the missing person with dementia, care partner, Coordinator, and volunteers. Participants ‘walked through’ (i.e., simulated) each step of the Community ASAP process to obtain a detailed understanding of the Community ASAP system in action as well as the opportunity to try the interface themselves.

To assess the accuracy of the alerts, participants recorded alerts received, messages sent, and content of alerts using an *Alert Tracking Form*. To evaluate the usability of the interface, researchers observed participants’ interactions with and responses to the interface and participants completed the *Website Usability Questionnaire* [16]. This questionnaire is comprised of seven questions and uses a five-point Likert scale ranging from strongly agree, agree, neutral, disagree, and strongly disagree. Questions pertained to the ease of navigating the interface, aesthetics, and its organization (S1 Appendix). Usability was also evaluated through focus groups and participants’ email responses to open-ended questions. Participants reported what they liked best and least about the Community ASAP system, challenges and frustrations using the interface, and suggestions for improvement. In one location, additional focus group questions addressed police and search and rescue processes related to missing persons incidents as well as how Community ASAP could be integrated into existing search and rescue processes.

We used descriptive statistics (i.e., averages, percentages, and chi-squared tests) to analyze data collected using the *Alert Tracking form*, and *Website Usability Questionnaire*. Focus groups were digitally recorded, transcribed verbatim, and managed using NVIVO12 software. Conventional content analysis [17] guided our analysis of data generated through observations, focus groups, and email responses to open-ended questions.

## Results

A total of 70 participants were recruited across the three phases of development and evaluation of Community ASAP. Of these participants, 3 were persons living with dementia, 3 were older adults with no cognitive impairment, 5 were care partners for someone living with dementia, 16 were from community organizations, and 28 were from police services. In this paper, we summarize and organize key results in three sections: 1) Concept of the system and its integration into existing police processes; 2) Interface usability and suggestions for improvements; 3) Experiences of partners during this iterative design process and in this study.

### Community ASAP concept and integration

#### Overall impressions of Community ASAP system

Participants stated that Community ASAP was a novel concept and a useful tool to help community members locate persons living with dementia who become lost. It could result in having more ‘eyes on the ground’, which could reduce the time and resources required from police services, noting:

If there are people that happen to be driving by…they will look out the window and see that person walking by. That’s a huge asset and that can prevent us from having to search all night long.

(Police 1, Phase II)

However, participants also identified the need to ensure that volunteers do not become actively involved in searching for the missing person as it can interfere with organized search processes and divert police resources away from the search.

#### Coordinator role in a police service

Participants agreed that in many Canadian jurisdictions, the role of the coordinator should be embedded within a police service. The reason is three-fold: 1) missing persons reports and searches are coordinated by police; 2) the Community ASAP system must be coordinated 24 hours per day, 7 days per week and in Canada, police organizations are one of the few services that provide this continuity of service; and 3) some police organizations dedicate an office to manage social media content so the addition of the monitoring of Community ASAP to this position is appropriate. However, participants also acknowledged that police services are driven by real-time events. When another major event occurs, a substantial number of officers may need to be allocated to the emerging situation.

Police participants explained that even though there was a good fit between the requirements of the coordinator and existing police processes, the Community ASAP concept was strong, and the system could be useful to police, Community ASAP must integrate or fit seamlessly with the existing software infrastructure.

[The] red flag potentially for me would be introducing a third-party app into the technical infrastructure that also introduces a weakness depending on the security set-up

(Police 3, Phase II)

#### Threshold for alerts

Participants from police and community organizations as well as those living with dementia liked that Community ASAP was independent of social media networks (Twitter, Facebook). Therefore, Community ASAP did not contain extraneous, irrelevant information like other social media do (e.g., advertisements). Community ASAP was developed specifically as a community response to occurrences of persons living with dementia going missing and thus contained only information specific to helping locate lost persons. This made it viable for people who did not use social media. Notably, participants were clear that a risk threshold, set by the local police organization, would need to be met prior to activating Community ASAP and sending an alert to volunteers. However, this threshold could be lower than when issuing a missing person alert to subscribers of police Facebook and Twitter feeds:

The threshold for us to do our social media really is higher than what [it would be] for Community ASAP. I thought this could go half-way between so we would probably generate five-fold the number of releases than what we do on social media

(Police 2, Phase II)

#### Release of information to volunteers

Some participants expressed concern about releasing information about missing persons living with dementia to volunteers who registered with Community ASAP. They identified the potential misuse of information released through Community ASAP for criminal purposes, putting a vulnerable person in danger. These participants indicated that the nature of the information to be released would have to be carefully considered as well as the risks inherent in the event (e.g., weather conditions, need for medications, vicinity to environment hazards, time missing).

A lot of people will prey upon our vulnerable population. We see it all the time and always look at a criminal element in everything that we do. The information that’s been shared to a whole bunch of volunteers can easily be taken and used so I think we have to be careful and be careful how much information we are sending to the volunteers.

(Police 3, Phase II)

While some participants suggested a screening and vetting process could be implemented prior to coming on board as a volunteer, others stated this is not feasible and that information about missing persons are already being released on social media and using conventional methods (e.g., TV, radio) to the general public without screening of recipients.

### Interface usability and suggested improvements

#### Accuracy

In this paper we report on the accuracy and usability of the Community ASAP system and interface in the final development and evaluation phase. A total of 110 alerts were initiated in the final development phase. Of these, 108 (98%) were received by participants. The two that were not received were due to an unstable internet connection and to a device that was turned off. This indicates that the Community ASAP system is functional and sends and receives alerts as expected.

#### Usability

Overall, participants found Community ASAP to be easy to learn and use, and all participants thought that they could see themselves becoming volunteers.

I could see myself becoming a community volunteer. It’s really simple, it doesn’t have a bunch of overhead for time. You just sign up and are notified when someone is missing in your area. If you could get enough people doing this it would make finding missing persons much faster.

(Community organization 1, Phase II)

**Fig 3:**
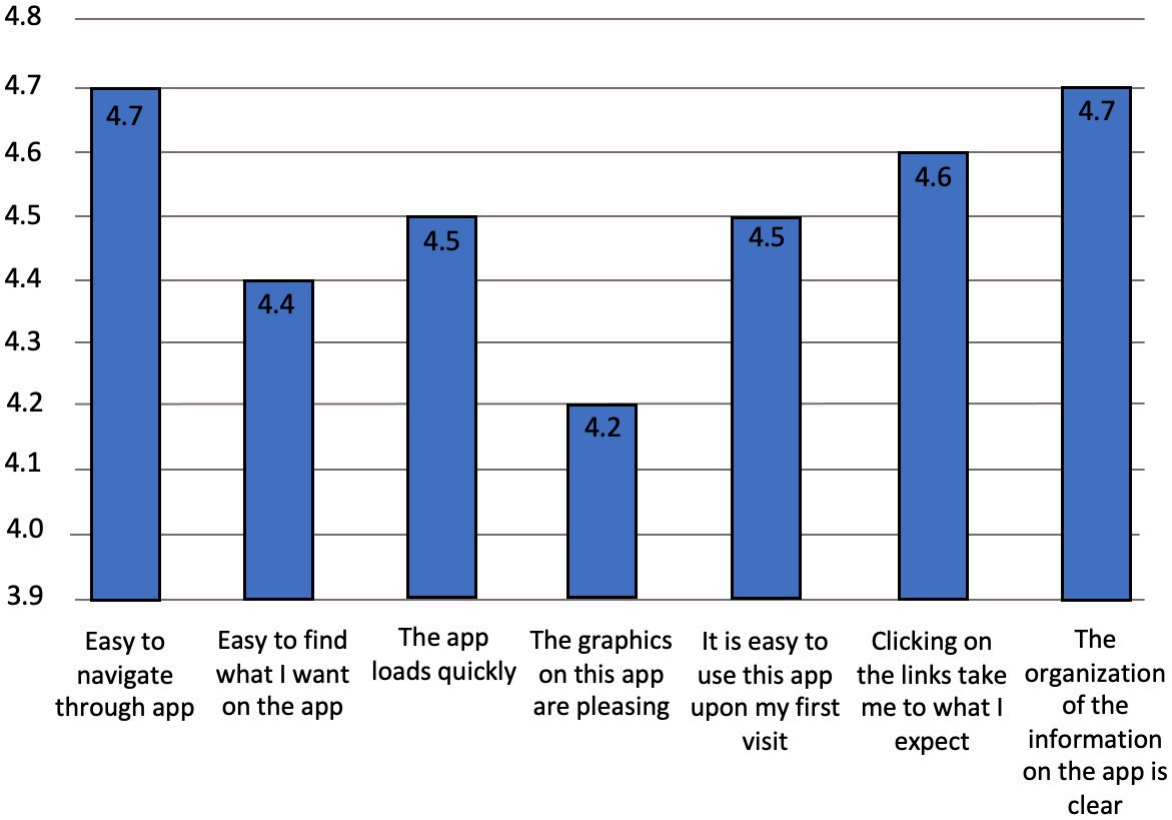
Results from Website Usability Questionnaire.

According to results from the Website Usability Questionnaire, participants thought the Community ASAP mobile application interface was easy to navigate; the graphics on the app were pleasing to the eye and did not load slowly; clicking on the links took them to where they expected; it was easy to find what they wanted on the app; the app was easy to use; and the organization of information on the interface was clear (Fig 3). A Chi-square test of independence was performed to examine the relationship between the scores derived from the Website Usability Questionnaire and the different phases of development. The relationship between these variables was significant, *x*^2^ (16, *N* = 441) = 96.0, *p* < 0.05. There was an increase in scores as the phases of development progressed.

#### Suggestions for improvements

Having seen the Community ASAP interface in action through participation in various ‘walk through’ simulations, participants had specific as well as general suggestions for improvements. Specific suggestions included the appearance of alerts and their linkage to other pages in the interface, navigation functions in the home page, selection and editing of alert channels, and use of metric as well as imperial units of measurement when describing the missing person. Suggestions highlighted the need to provide comprehensive instructions and help features within the interface. They also suggested a demonstration video and resource for technical support.

People need to have training or support. Maybe a demo video…need to be able to support those who are not experienced users.

(Older adult 1, Phase III)

For Community ASAP to have an impact on the lives of persons with dementia who are missing and their care partners, it would need to be downloaded and used widely. For this to occur, trouble-shooting support must be available and easily accessible, particularly for those volunteers who have lower technological literacy than volunteers who ‘grew up’ using tablets and smart phones.

Participants also identified the need to improve how alerts are presented. For example, one participant described that he put his phone face down shortly before the simulation began and did not realize that he received an alert until approximately 45 minutes later when he picked up his phone and realized that he had missed four alerts. He realized that this was because the Android phone was face down which makes alerts inaudible. As volunteers may receive many notifications from different software and apps, other participants suggested that Community ASAP alerts should have unique sounds that are controlled by the user so that they can be easily distinguished from others.

Participants recommended that the colour scheme be changed to improve aesthetics of the interface and readability and usability.

Too much gray and white. Should be black and white. It’s difficult to distinguish the foreground from the background.

(Person living with dementia 1, Phase III)

Another participant stated that while icons can be helpful for those with poor vision or English literacy, all icons should be supplemented with key words as icons can be misinterpreted.

Through successive simulations, it became evident that participants made errors when entering their information into the Community ASAP interface, perhaps due to using smart phones which have limited screen size. A participant suggested adopting a Master Name Index to prevent such data errors. This Index requires users to enter the name only once. Other fields are populated from the Index and when a correction is made in the Index, associated fields are automatically corrected.

### Experiences of partners during Community ASAP design and evaluation

Participants reported that taking part in the design and evaluation of Community ASAP afforded them the opportunity to collaborate with and learn from those in different organizations and sectors (i.e., police, health care, social services, research) for the ultimate purpose of informing a community response to the issue of persons living with dementia who go missing. While these participants may have worked and interacted with other organizations prior to their involvement in Community ASAP, they did not necessarily understand the depths of issues, perspectives, and processes within other organizations until engaging in the simulation with other partners.

This experience has been amazing. They [the public] really have no idea or the number of resources that you [the police] put into a person when they go missing. They think it’s a police car driving around the neighbourhood. The word has to get out there that it’s very different than what they are perceiving. And its probably part of our job as the education so that they see that there is this whole community approach from first responder to education and together that is what makes a difference out there for people who are vulnerable.

(Community organization 2, Phase II)

This resulted in mutual respect as well as deeper and stronger relationships between partners and their work toward their re-affirmed common goal. The trust that was built between partners and the new relationships that were established can be harnessed to mobilize partners toward continued action toward the goal of creating a safety net for persons living with dementia who become lost.

The work of this group started around the idea of Community ASAP and it was just a glimmer in the mind of the person who thought of the idea of Community ASAP. But look at where we are. Whether or not we use Community ASAP, we’ve just spent the day together. Police, community, researchers talking about this issue which is amazing.

(Community organization 1, Phase II)

## Discussion

The purpose of this study was to develop and evaluate the accuracy and usability of a community-based alert system that allows first responders to initiate alerts of missing persons with dementia to community volunteers. This is the first study to focus on and describe the process of developing a community-based alert system of this nature in Canada. To our knowledge, there is no system or mobile application like Community ASAP in Canada. Overall, the outcomes from this study were positive. Participants stated they could see themselves becoming community volunteers using Community ASAP. Many thought the app was easy to use, and participants, such as those from police organizations believed that by having more ‘eyes in the community’ would potentially alleviate the time and resources required from their services. The iterative design process enabled participants to provide feedback over the span of three phases of development, which contributed to Community ASAP’s evolution.

The successes of Community ASAP were largely attributed to the walk-through simulations that took place with local police and community members. As Amber Alert is the only community wide alert system available in Canada [18], it was unknown prior to the project whether a community-based alert system for missing persons living with dementia, such as Community ASAP, could be hosted within policing organizations. During the simulations with different police services, it became evident that Community ASAP must be moderated by police services; they are essential in issuing alert due to their direct involvement in missing persons calls and their responsibility to safeguard the person’s wellbeing. Adults generally have the legal right to be missing, to leave their place of normal residence and cut off contact with those around them [19]. It is a police organization’s responsibility to determine whether they need to respect that person’s right to confidentiality over their whereabouts, or if there are legal grounds to forcibly return them to their residence. As police organizations receive missing person reports, situating Community ASAP within police services is an appropriate process in the Canadian context.

Community ASAP if adopted within police services can serve as a strategy to pave the implementation of ‘community policing’ to assist in the management of missing person incidents. Community policing is a strategic innovation that involves at least three organizational stances, i.e., involvement of the community, decentralization of the services, and the adoption of problem-solving orientation services [20]. We believe that Community ASAP could contribute by involving members of the community and assist in implementing a problem-solving approach in police departments when a person living with dementia is missing. Community policing has been defined as ‘the efforts to develop partnerships with groups and individual community members’ [20]. These partnerships involve relationships between police and other organizations that have direct responsibility for the quality of neighborhood life such as the schools and agencies responsible for health. Community ASAP can be an example that materializes the idea of police and members of the community being ‘co-producers’ (i.e., the cornerstone of the community policing) of safety of persons living with dementia at risk of getting lost. The problem-solving approach follows a proactive approach as it focused on discovering the situations that produce calls for police assistance (in this case a missing person), identifying the causes that lie behind them, and designing and implementing strategies to deal with these causes. For example, with the data about incidents stored in Community ASAP, problem-oriented policing can be used in this specific context as an analytic method for developing solutions when a missing person incident occurs.

The simulations embedded within this project also enabled the project team to identify precisely how Community ASAP needed to be developed to fit within the police service’s existing infrastructure. We did this by ‘walking’ all participants, representing multiple organizational and departmental perspectives, step-by-step through the process of locating a missing person, from the first step of receiving a report to the final steps involved in locating the person, and hopefully returning the person safely home. This enabled the research team to gain a clearer understanding as to where the integration of Community ASAP would fit within existing standard operating procedures among police and volunteer search and rescue services in Canada. While simulations have become common practice in the education of police officers [21, 22], and in the evaluation of technologies for search and rescue such as drones [23], there is limited evidence to support the use of ‘walk-through’ simulations to evaluate community-based alert systems. The process of facilitating walk-through simulations with stakeholders such as police and community organizations could be used as a model for future projects that involve the development of alert systems.

In addition to the ‘walk-through’ simulations, integrating multiple phases of usability testing and following approaches similar to that of design-centered entrepreneurship [24] was also integral in the development of the Community ASAP system. In the first phase, in-the-field scenarios were conducted to evaluate the prototype web-based system’s function and usability. The live simulation of the pilot version of Community ASAP allowed the research team to understand the concept that was embedded within the alert system by internal partners sharing challenges, frustrations and suggestions for improvement of the system. The second and third phases facilitated additional simulations of the web-based system and mobile application among potential end users. As a result, all three phases allowed Community ASAP to move from proof of concept to the finalized product that is now ready for commercialization or deployment.

While it was recommended that police services should be responsible for initiating community alert systems such as Community ASAP in Canada, this approach may not apply in other areas of the world. Purple Alert [25], in Scotland, is a free mobile application that uses similar concepts as Community ASAP; an alert is initiated to subscribers of the app when a person with dementia is reported missing. Since its initial release in 2017, Purple Alert has received more than 10,000 downloads. Rather than having the alert initiated by the police, designated staff within Alzheimer Scotland moderate the system and issue alerts [26]. Like Community ASAP, Police Scotland and other partners were present throughout the development process, to ensure the app and the service aligned with the existing infrastructure. Other countries interested in integrating a community-based alert system designed for lost persons with dementia, should consider the capacity and available resources of police and community members in their region to ensure the most appropriate fit of the alert system.

A participatory approach in the design, development, and evaluation of Community ASAP was vital in ensuring the system was developed in a way that would enable quick uptake by end-users [27]. By involving multiple stakeholders in the walk-through simulations, and engaging in continuous communication throughout the project’s lifespan, we helped participants gain a deeper understanding of each stakeholder’s roles and responsibilities [28] as it relates to lost and missing persons with dementia and ensured Community ASAP was co-created by the targeted end-users. The involvement of first responders and community organizations across all phases of this project improved the outcomes of this project and enhanced the working relationships among involved stakeholders in a way that has sparked other initiatives. For example, we collaborated with the Alzheimer Society of Ontario to complete a study on the need to educate police services on best practices to reduce the time it takes to locate and return home missing persons with dementia. Police and academic collaborations have the capacity to bring about new ways of thinking and acting within police organizations through participatory approaches [29]. The relationships mobilized in this project will stimulate incremental changes among police services that are necessary to mitigate the risks and adverse outcomes when persons living with dementia become lost.

The privacy of missing persons was brought up as a concern among participants. On one hand, a certain amount of information is needed for community volunteers to be able to identify a person as fitting the descriptions of a person reported missing. On the other hand, certain details, such as notifying the public that the missing individual has a cognitive impairment, may make the individual more vulnerable to crimes. Although participants did not bring up concerns about volunteers’ privacy, this may become a barrier to actual use by community citizens. Future volunteers may wish to know what happens to their personal data such as email, mobile phone number and preferred geographic radius which references their locations.

## Conclusions

Three phases of development and evaluation were completed on the usability and functionality of a community alert system for missing persons with dementia (Community ASAP). Participants found Community ASAP to be accurate easy to learn, use, and navigate, and was feasible to operate across all phases of development and evaluation. Individual interviews and focus group discussions showed that the system allowed community volunteers to participate by providing extra eyes on the ground while first responders conduct their searches. Suggestions for future improvements include features to make the usability of the interface including adding instructions such as a demonstration video and technical support within the app, improving the navigation within the home screen, and improving the visual presentation of the app such as the colour scheme.

As the development and research phases of the initiative have been completed, Community ASAP deployment is now under the direction of its business owner.

## Supporting information

S1 Appendix (DOCX)
