# Supplementary material for: Mobile alert app to engage community volunteers to help locate missing persons with dementia
Source: PLoS One. 2021 Jul 19;16(7):e0254952. doi: 10.1371/journal.pone.0254952 (PMC8289012; doi:10.1371/journal.pone.0254952)
Supplement: S1 Appendix — (DOCX) [file pone.0254952.s001.docx]

**S1 Appendix**

**Alert Tracking Form**

**Volunteer Code:**

***PREFERENCES***

**Area(s) of availability:**

**Radius:**

**Notification method:**

**Notification of missing person received?**  Yes No

**Time received (if applicable): ______________________**

**Received via:**  Text Email Text and email

**Found/closing report received?**  Yes No

**Time received (if applicable): ______________________**

**Received via:**  Text Email Text and email

**Website Usability Questionnaire**

Date: _________ Participant code: __________

**Consider each statement and select your agreement with each one**

1. **It is easy to navigate through this website**

Strongly Agree

Agree

Neutral

Disagree

Strongly Disagree

1. **It is easy to find what I want on this website**

Strongly Agree

Agree

Neutral

Disagree

Strongly Disagree

1. **This website loads too slowly**

Strongly Agree

Agree

Neutral

Disagree

Strongly Disagree

1. **The graphics on this website are pleasing to the eye**

Strongly Agree

Agree

Neutral

Disagree

Strongly Disagree

1. **It is easy to use this website upon my first visit**

Strongly Agree

Agree

Neutral

Disagree

Strongly Disagree

1. **Clicking on the different sections takes me to what I expect**

Strongly Agree

Agree

Neutral

Disagree

Strongly Disagree

1. **The organization of information on the system screen is clear**

Strongly Agree

Agree

Neutral

Disagree

Strongly Disagree

**Mobile Phone Usability Questionnaire**

Date: _________ Participant code: __________

**Consider each statement and select your agreement with each one**

1. **It is easy to navigate through this application**

Strongly Agree

Agree

Neutral

Disagree

Strongly Disagree

1. **It is easy to find what I want on this application**

Strongly Agree

Agree

Neutral

Disagree

Strongly Disagree

1. **This application loads too slowly**

Strongly Agree

Agree

Neutral

Disagree

Strongly Disagree

1. **The graphics on this application are pleasing to the eye**

Strongly Agree

Agree

Neutral

Disagree

Strongly Disagree

1. **It is easy to use this application upon my first visit**

Strongly Agree

Agree

Neutral

Disagree

Strongly Disagree

1. **Clicking on the different sections takes me to what I expect**

Strongly Agree

Agree

Neutral

Disagree

Strongly Disagree

1. **The organization of information on the system screen is clear**

Strongly Agree

Agree

Neutral

Disagree

Strongly Disagree
